# Challenging for cartilage repair

**DOI:** 10.1186/1758-2555-1-13

**Published:** 2009-07-14

**Authors:** Mitsuo Ochi

**Affiliations:** 1 Department of Orthopaedic Surgery, Graduate School of Biomedical Sciences, Hiroshima University, Hiroshima, Japan

## Editorial

When we look back on the past 15-year history of cartilage repair, it is clear that remarkable progress has been made in this area. There is no doubt that a lot of studies have been carried out on cartilage repair and chondrocytes since Brittebergs' report on ACI in 1994 [1]. I would like to introduce our multi-pronged approach to cartilage repair. After the 1994 report [1], we performed implantation of tissue-engineered cartilage made ex vivo for the treatment of osteochondral defects of the joints, to avoid the leakage of grafted cultured chondrocytes in suspension [2,3]. Sixty knees of 57 patients with full-thickness cartilage defects were followed-up over 5 years. The clinical rating improved significantly after implantation of tissue-engineered cartilage and was maintained for an average of 8.3 years. The arthroscopic findings 2 years after implantation were graded as normal or nearly normal according to the ICRS scale in more than 90% of patients. Biomechanically, stiffness of the graft almost equaled the surrounding normal cartilage (87.9 ~102.5%) 2 years after implantation.

The next contribution to our variety of cartilage repair methods, was the minimally invasive approach using a tissue-engineered chondral plug. New scaffolds, consisting of a type I collagen sponge and surrounding PLLA mesh [4] or interconnected porous calcium hydroxyapatite ceramic [5], were demonstrated to comprise an effective minimally invasive approach.

The bone marrow stimulating technique under arthroscopy is another minimally invasive technique which is a well accepted procedure for large osteochondral defects. However, there are two potential weak points when inducing hyaline cartilage. One is a compressive overload on the drilled or microfractured area in the early stage after surgery. In order to reduce this early stage overload, we have developed external fixators which allow almost full ROM with joint distraction for clinical cases, based on an animal study [6]. This apparatus has been successfully used for 12 patients, each with a large cartilage defect [7]. Another weak point is a small number of mesenchymal stem cells obtained from drilled holes which are used for chondrogenesis. An injection of cultured MSCs into the joint has been demonstrated to be effective for cartilage defects in rats [8]. The combined approach should be adopted for large defects in the near future.

However, the most optimal procedure for the repair cartilage defects is simply an injection of cytokines or growth factors and cells. Our planned novel approach for the future is to use a cell delivery system using an external magnetic field. Our procedure involves using autologous bone marrow mesenchymal stem cells attached to small-sized magnetic beads and an external magnetic field. For successful cartilage repair, it is ideal to effectively attract injected mesenchymal stem cells to a desired portion in the knee joint (osteochondral defect) using an external magnet force [9]. We believe that this novel system is also effective in the treatment of brain or spinal cord injury and for malignant tumors, using natural killer cells instead of autologous bone marrow mesenchymal stem cells.

Current clinical results following cartilage repair remain unsatisfactory. We need to perform several prospective studies, in order to obtain solid evidence from each procedure's individual indication. From this, decisions can be made to enhance cartilage regeneration with hyaline cartilage. However, continuous efforts in experimental research, based on completely novel and challenging ideas, are vital in order to achieve a breakthrough in cartilage surgery. I hope that such a breakthrough will come soon.
